# Ghrelin Ameliorates Diabetic Retinal Injury: Potential Therapeutic Avenues for Diabetic Retinopathy

**DOI:** 10.1155/2021/8043299

**Published:** 2021-10-26

**Authors:** Jie Bai, Fan Yang, Ruiqi Wang, Qinghui Yan

**Affiliations:** ^1^The Fourth Affiliated Hospital, Zhejiang University School of Medicine, N1 Shangcheng Road, Yiwu, Zhejiang, China; ^2^The First Affiliated Hospital, Harbin Medical University, 23 Youzheng Street, Harbin, Heilongjiang, China; ^3^Beijing Min Zhong Eye Hospital, Lincui Road, Chaoyang District, Beijing, China

## Abstract

Ghrelin has anti-inflammatory, antioxidant, and antiapoptotic effects, and it may be beneficial for the treatment of many ophthalmic diseases, such as cataract, uveitis, and glaucoma. Our previous work proved that ghrelin pretreatment reduced the apoptosis of lens epithelial cells induced by hydrogen peroxide, reduced the accumulation of reactive oxygen species (ROS), and effectively maintained the transparency of lens tissue. However, no study has yet investigated the effect of ghrelin on retina. In this study, we conducted in vitro and in vivo experiments to explore the effect of ghrelin on high-glucose- (HG-) induced ARPE-19 cell damage and diabetic retinopathy in streptozotocin-induced diabetic rats. ARPE-19 cells were incubated in a normal or an HG (30 mM glucose) medium with or without ghrelin. Cell viability was measured by 3-(4, 5-dimethylthiazol-3-yl)-2,5-diphenyl tetrazolium bromide assay, and apoptosis was detected by the Hoechst–PI staining assay. Intracellular reactive oxygen species (ROS) production levels within cells were measured using 2′,7′-dichlorofluorescein diacetate staining, and the contents of superoxide dismutase and malondialdehyde were measured using relevant detection kits. The expression levels of IL-1*β* and IL-18 were measured using an enzyme-linked immunosorbent assay, and those of NLRP3, IL-1*β*, and IL-18 were measured using Western blotting. The rat diabetes models were induced using a single intraperitoneal injection of streptozotocin (80 mg/kg). The morphological and histopathological changes in the retinal tissues were examined. The results indicated that ghrelin reduced ROS generation, inhibited cell apoptosis and the activation of NLRP3 inflammasome, inhibited the apoptosis of retinal cells in diabetic rats, and protected the retina against HG-induced dysfunction. In conclusion, ghrelin may play a role in the treatment of ocular diseases involving diabetic retinopathy.

## 1. Introduction

With the rapid development of the social economy and the change in people's lifestyle and eating habits, the prevalence of diabetes is also rising. Diabetic retinopathy (DR) is the most common microvascular complication of diabetes. Its incidence rate is positively related to the course of the disease [1, 2]. Studies have shown that 50% of patients with diabetes for more than seven years have DR. Under physiological conditions, the tight junction between retinal capillary endothelial cells and the retinal pigment epithelial cells constitute the blood–retinal barrier, which can block the damage of some harmful components (e.g., lymphotoxins, inflammatory factors, and immune complexes) in the blood to the retina [3, 4]. Long-term hyperglycemia and changes in blood components in diabetic patients destroy the blood–retinal barrier and cause retinal capillary pericyte necrosis and endothelial dysfunction, leading to the leakage of liquid components in the blood vessels into the retinal space. This causes a series of changes in the retinal tissue, such as bleeding, edema, exudation, and ischemia [5]. Although anti- Vascular endothelial growth factor (VEGF) drugs and hormone drugs have been used in the treatment of DR in recent years, the treatment goal is mainly for diabetic macular edema, which requires long-term continuous administration. Moreover, vision can be improved during treatment, but once treatment is stopped, recurrence of macular edema easily occurs [6]. This treatment method cannot fundamentally solve the problem of diabetic retinal injury. Therefore, to explore early molecular biological changes in DR and to find potential therapeutic methods are of great clinical significance.

DR has had varying degrees of impact on the lives and work of patients. Therefore, controlling diabetes and DR has great strategic significance in the health level and economic interests of people in China and worldwide. DR has been proven to be closely related to oxidative stress and chronic inflammation. Inflammatory cytokines are highly expressed in the retinas of DR patients, promoting the occurrence and development of DR [7, 8]. Therefore, protecting retinal pigment epithelial (RPE) cells and reducing the influence of pathogenic factors on the degeneration and functional decompensation of RPE cells play an important role in the prevention, treatment, and remission of DR.

The growth hormone-releasing peptide (ghrelin) has many biological activities, such as heat generation, improving the survival rate of neurons, regulating apoptosis, minimizing anti-inflammation, and enhancing immunity and anti-oxidation. Ghrelin has been proven to be an effective drug for many diseases, including ischemia reperfusion, gastrointestinal stromal tumors, diabetic neuropathy, myocardial infarction, and lung injury [9, 10]. In our previous work, ghrelin pretreatment was shown to reduce the apoptosis of lens epithelial cells induced by hydrogen peroxide, reduce the accumulation of reactive oxygen species (ROS), increase the expression of superoxide dismutase (SOD) and catalase, and decrease the expression of malondialdehyde (MDA). These results suggest that ghrelin can inhibit the oxidative damage and apoptosis of ciliary epithelial cells by preventing the damage of lens membrane lipid. In addition, morphological observation results have shown that ghrelin can effectively maintain the transparency of lens tissue, providing a new drug option for the treatment of cataract [11].

As ghrelin has anti-inflammatory, antioxidant, and anti-apoptotic effects, it may be beneficial for the treatment of many ophthalmic diseases, such as uveitis, cataract, and glaucoma. At the same time, ghrelin may have a positive effect on retinal ganglion cells (RGCs), Muller cells, and RPE cells, and it may have a protective effect on the retina, and it may be a new way to treat retinopathy. No study has yet investigated the effect of ghrelin on DR. In this study, we conducted in vitro and in vivo experiments to explore the effect of ghrelin on DR and to elucidate the protective effect of ghrelin.

## 2. Materials and Methods

### 2.1. Chemicals

Ghrelin (Sigma-Aldrich, St. Louis, MO, USA) was dissolved in dimethyl sulfoxide (DMSO, Solarbio, Beijing, China) and stored at −20°C for later use. The following were also used: Dulbecco's Modified Eagle Medium (DMEM), 3-(4, 5-dimethylthiazol-3-yl)-2,5-diphenyl tetrazolium bromide (MTT), 2',7'-dichlorofluorescein diacetate (H_2_DCFDA) (Solarbio, Beijing, China), Hoechst–PI staining kit, SOD and MDA assay kits (Nanjing Jiancheng Bioengineering Institute, Nanjing, China), IL-1*β* and IL-18 enzyme-linked immunosorbent assay (ELISA) kits (Shanghai Westang Biotech Co., Ltd.), and bicinchoninic acid (BCA) protein assay kit (Beyotime Institute of Biotechnology, Shanghai, China).

### 2.2. Cell Culture

The ARPE-19 cells were obtained from the American Type Culture Collection, cultured in DMEM in a humidified atmosphere with 5% CO_2_ at 37°C, and supplemented with 10% fetal bovine serum, 100 U/mL penicillin, and 100 *μ*g/mL streptomycin. To test the effect of ghrelin on high-glucose (HG) (30 mM glucose)-induced cell viability, the ARPE-19 cells were treated with ghrelin (1 *μ*M), followed by HG, for three days.

### 2.3. MTT Assay

The viability of cells was measured by MTT assay. A total of 50 *μ*L MTT (5 mg/mL in PBS) was combined with 450 *μ*L of culture medium from each well and incubated for 4 h. The absorbance value was measured using a microplate reader at 490 nm.

### 2.4. Hoechst–PI Staining Assay

The cells were resuspended by adding 5 *μ*L Hoechst 33342 staining solution (10 *μ*g/mL) and 5 *μ*L PI staining solution (10 *μ*g/mL). The cells were incubated at 4°C for 20 min, washed with PBS, and captured under a fluorescence microscope.

### 2.5. Basal ROS Level

The ROS production levels within the RPE cells were measured using H_2_DCFDA staining at 37°C for 30 min and then washed twice with PBS. Fluorescence changes were measured using fluorescence spectrophotometry at 485 nm (excitation)/535 nm (emission).

### 2.6. Measurement of SOD and MDA

The total SOD and MDA activities were measured using relevant detection kits. Briefly, the cells were collected, and the lysates were prepared using ultrasound at an amplitude of 24 kHz for 2 s and then centrifuged further for 10 min at 12,000 × *g* at 4°C. Absorbance was measured at 532 nm and 450 nm.

### 2.7. ELISA Analysis of IL-1*β* and IL-18 Release

The supernatants of the ARPE-19 cells were extracted through centrifugation at 1,000 g for 10 min. The expression levels of IL-1*β* and IL-18 were measured using an ELISA kit according to the manufacturer's instructions.

### 2.8. Western Blot Assay

The cells were collected, and the lysates were centrifuged at 14,000 RPM at 4°C for 5 min. The BCA kit was used to determine the concentration of tissue protein. The proteins were separated by 10% SDS-PAGE, transferred to a non-fat milk blocked PVDF membrane for 1 h, and incubated with anti-NLRP3 (1 : 500; Santa Cruz Biotechnology, USA), anti-IL-1*β* (1 : 500 dilution; Santa Cruz Biotechnology, USA), and anti-IL-18 (1 : 500 dilution; Santa Cruz Biotechnology, USA). Blots were visualized with an ECL Western blot detection reagent (Pierce Biotechnology). The band intensities were calculated using ImageJ software.

### 2.9. Animals and Treatment

#### 2.9.1. Animals and Group

Male Wistar rats weighing 220–280 g were obtained from the Second Affiliated Hospital of Harbin Medical University (laboratory animal license number: SCXK [Heilongjiang Province] 2019-012) and raised with a 12/12 h light–dark cycle in a temperature-controlled facility (humidity: 60%, temperature: 23–25°C). Their weights were measured once a week. The animal experiments were approved by the Institutional Animal Care and Use Committee of Harbin Medical University and carried out in accordance with the National Institutes of Health Guide for the Care and Use of Laboratory Animals (NIH Publications No. 8023, revised 1978). The rats were adaptively fed in the Experimental Animal Center for seven days and then divided into the control group (*n* = 6), the ghrelin group (*n* = 6), the HG group (*n* = 6), and the HG + ghrelin group (*n* = 6) using a random number table. The rats in the control group were normally fed without any treatment. The rats in the HG group received 1% streptozotocin solution (60 mg/kg) once by intraperitoneally injection, and those with blood glucose >16.7 mmol/L were selected as diabetic rats. The rats in the HG + ghrelin group were given ghrelin (100 *μ*g/kg/day) once a day through subcutaneous injection for four weeks after being injected with streptozotocin through intraperitoneal injection. The rats in the control group received a single intravenous injection of 0.9% NaCl. Tail vein blood was taken for blood glucose and HbA1c detection.

#### 2.9.2. Retinal Hematoxylin–Eosin Staining

The rats were euthanised by intraperitoneal injection of pentobarbital sodium (200 mg/kg), eye balls were enucleated and fixed in 4% paraformaldehyde for 24 h, the ocular anterior segments were resected, the entire retina was sliced along the sagittal plane and the sections were dehydrated with gradient ethanol and washed with distilled water three times. The slices were stained with Harris hematoxylin for 3-8 min, washed with tap water, stained with eosin dye for 1–3 min, dehydrated with gradient ethanol again, rendered transparent with xylene, and observed using light microscopy. The thickness of the outer nuclear layer (ONL) and inner nuclear layer (INL) was measured using the ImageJ image processing program.

#### 2.9.3. TUNEL Assay

The paraffin sections were routinely dewaxed to hydration, and 0.25% Triton X-100 sodium citrate solution (0.1%) was reacted at 4°C for 30 min. The TUNEL reaction mixture was prepared according to the kit's instructions. Then, 50 *μ*L peroxidase was added, and the sections were incubated at 37°C for 30 min, the rate of apoptosis in the RGC layer in each group was calculated.

#### 2.9.4. Retinal Vessels

The retinas were separated and washed with distilled water. The internal limiting membrane, with a vitreous body on the surface, was avulsed and divided into three pieces, with the optic disc as the center. The retina was incubated in a 3% trypsin solution at 37°C for 3 h. The retina was placed in distilled water, and the nerve tissue was removed by gently blowing with a straw. Retinal vascular patches were mounted on slide and soaked in distilled water for 5 minutes, after periodic acid-Schiff (PAS) staining at room temperature for 15 minutes, the slide was rinsed in dH_2_O and stained with hematoxylin for 20 minutes, dehydration with an ethanol gradient, cleared with xylene, and observed under light microscope. The retinal vessels were red, and the nucleus was blue.

### 2.10. Statistical Analysis

All experiments were performed in triplicate, and the data were expressed as the mean ± standard deviation. One-way analysis of variance (ANOVA) was used for statistical analysis using GraphPad Prism 6.0 (GraphPad Software, Inc., USA). A *P* value < 0.05 was considered statistically significant.

## 3. Results

### 3.1. Ghrelin Protects Cell Viability and Prevents HG-Induced Cell Death

**Figure 1:**
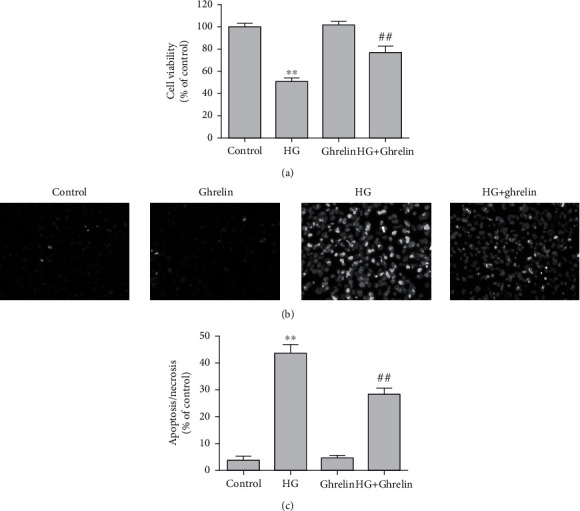
Ghrelin increases cell viability and reduces the apoptosis of ARPE-19 cells exposed to HG. (a) Cell viability was measured using MTT assay. (b) Cell staining imaged by a fluorescence microscope after Hoechst 33342 (blue) and PI (red) staining (×200). (c) Quantitative analysis of Hoechst–PI staining. ^∗∗^*P* < 0.05 vs. control group; ^##^*P* < 0.05 vs. HG group. Scale bar: 20 *μ*m.

The MTT assay revealed that HG caused a marked reduction in cell viability. In comparing the control group and the ghrelin group, ghrelin treatment significantly prevented cell loss (Figure 1(a)). Hoechst–PI staining assay was used to observe RPE cell apoptosis. The cells in the control and ghrelin groups showed normal nuclear morphology, with uniform light blue nuclei. Conversely, the cells in the HG group were shrunken with condensed chromatin. The nuclei of apoptotic cells were bright blue, and the cells in bright red (necrotic nucleus) clearly increased. In the HG + ghrelin group, the number of apoptotic and necrotic nuclei was significantly decreased (Figures 1(b) and 1(c)).

### 3.2. Ghrelin Decreases ROS Production, Increases SOD Activity, and Decreases MDA Formation in ARPE-19 Cells under HG Conditions

**Figure 2:**
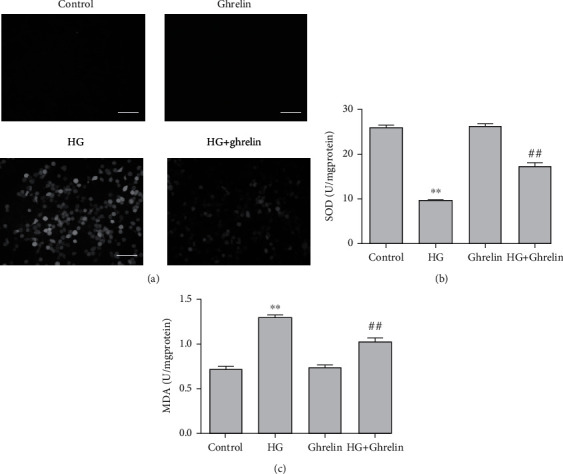
Ghrelin decreases ROS production, increases SOD activity, and decreases MDA formation in ARPE-19 cells under HG conditions. (a) Cellular ROS production was assessed using H_2_DCFDA staining. (b) SOD activity was assessed using biochemical assays. (c) MDA production was assessed using biochemical assays.^∗∗^*P* < 0.05 vs. control group; ^##^*P* < 0.05 vs. HG group. Scale bar: 20 *μ*m.

Enhanced oxidative stress was observed in the HG group. Ghrelin significantly attenuated ROS production in ARPE-19 cells cultured under HG conditions (Figure 2(a)). Treatment with ghrelin significantly increased SOD activity (Figure 2(b)) and suppressed MDA generation (Figure 2(c)).

### 3.3. Changes in IL-18 and IL-1*β* Levels in the Supernatants and Changes in the NLRP3, IL-1*β*, and IL-18 Protein Expressions in Cells

**Figure 3:**
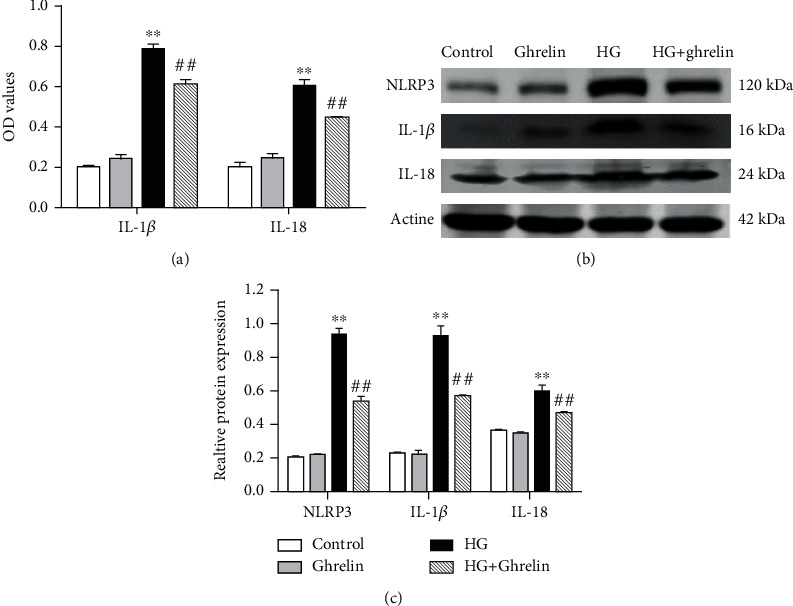
Measurement of inflammatory factor levels and protein expressions of NLRP3, IL-1*β*, and IL-18. (a) IL-1*β* and IL-18 levels in the cell supernatants of ARPE-19 cells were detected using ELISA. (b) The expressions of NLRP3, IL-1*β*, and IL-18 were detected using Western blotting. (c) Optical density ratios of NLRP3, IL-1*β*, and IL-18. ^∗∗^*P* < 0.05 vs. control group; ^##^*P* < 0.05 vs. HG group.

The activities of the IL-1*β* and IL-18 levels were detected using ELISA. The results showed that both IL-1*β* and IL-18 levels increased in the HG group and decreased in the HG + ghrelin group (Figure 3(a)). The protein expressions of NLRP3, IL-1*β*, and IL-18 were also examined within cells. HG treatment led to high levels of NLRP3, IL-1*β*, and IL-18 protein expressions, and ghrelin treatment reduced this expression (Figures 3(b) and (c)).

### 3.4. Effects of Ghrelin on Blood Glucose and HbA1c Levels

**Figure 4:**
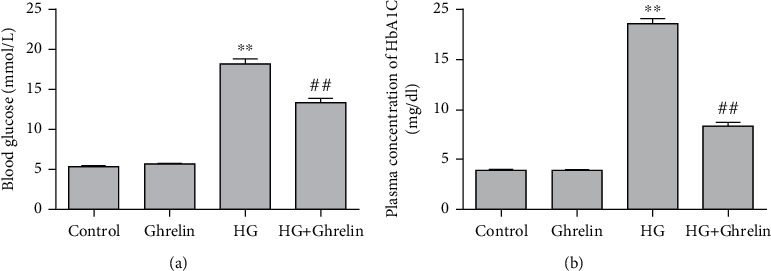
Effects of ghrelin on blood glucose and HbA1c levels: (a) blood glucose level and (b) HbA1c level.^∗∗^*P* < 0.05 vs. control group; ^##^*P* < 0.05 vs. HG group.

Compared with the control group, the rats in the HG group had high blood glucose and HbA1c levels. Treatment with ghrelin reduced blood glucose and HbA1c levels (Figure 4).

### 3.5. Ghrelin Inhibits Retinal Morphological Changes in Diabetic Rats

**Figure 5:**
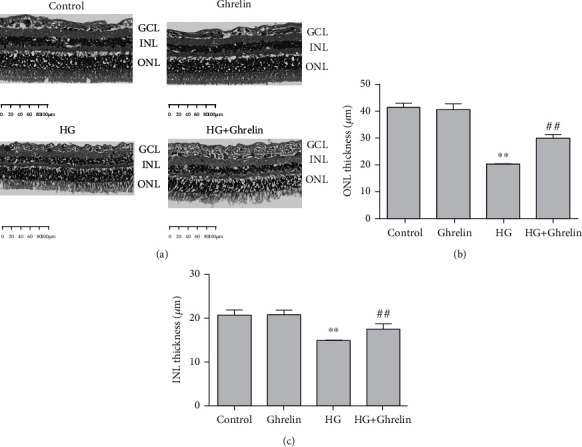
Histopathological examination of retina: (a) retinal HE staining, (b) quantitative analysis of ONL thickness, and (c) quantitative analysis of INL thickness. ^∗∗^*P* < 0.05 vs. control group; ^##^*P* < 0.05 vs. HG group.

In the control and ghrelin groups, the retinal structure showed orderly arranged retinal ONL and INL. In the model group, the arrangement of the inner and outer nuclear layers was slightly disordered, in the HG + ghrelin group, the histological changes were less pronounced. We also measured the thickness of the ONL and INL and compared the results of each group (Figure 5). In control group, the mean thickness of ONL and INL was 41.63 ± 1.82 *μ* m and 20.60 ± 1.17 *μ* m; In ghrelin group, the mean thickness of ONL and INL was 40.88 ± 2.10 *μ*m and 20.63 ± 1.01 *μ*m; in the HG group, we observed a remarkable decrease in ONL and INL thickness, and the mean ONL thickness was reduced by 50% (20.40 ± 0.57 *μ* m, *P* <  0.05) versus the control group, the mean INL thickness was reduced by 25% (14.57 ± 0.34 *μ* m, *P* <0.05) versus the control group. Administration of ghrelin significantly suppressed the thinning of ONL and INL thickness induced by streptozotocin solution, and the mean thickness of ONL and INL in HG + ghrelin group was 30.13 ± 1.33 *μ* m and 17.57 ± 0.93 *μ* m, respectively. Overall, the results revealed that ghrelin supplementation protects the function and structure of retina from HG-induced damage.

### 3.6. Ghrelin Inhibits Retinal Cell Apoptosis In Vivo

**Figure 6:**
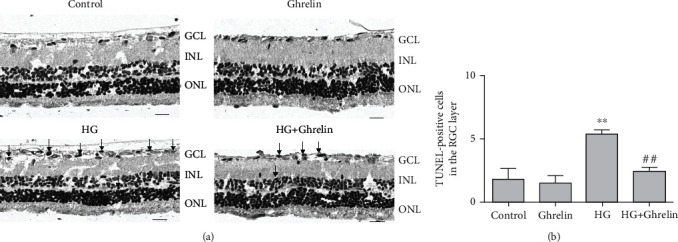
Ghrelin decreased apoptosis of GCL cells. (a) TUNEL staining of the retina. Black arrows show that the apoptotic cells were stained brown-yellow. (b) Quantitative analysis of TUNEL-positive cells. ^∗∗^*P* < 0.05 vs. control group, ^##^*P* < 0.05 vs. HG group. Scale bar: 30 *μ*m.

Brown-yellow staining of the nuclei represents cell apoptosis. There was no apoptosis in the RGC layer of the control and ghrelin groups. TUNEL-positive cells were significantly present in the RGC layer of the model group, and apoptosis occurred in the inner nuclear layer. TUNEL-positive cells in the HG + ghrelin group were fewer than those in the model group (Figure 6).

### 3.7. Ghrelin Attenuates Vascular Damage in the Retina

**Figure 7:**
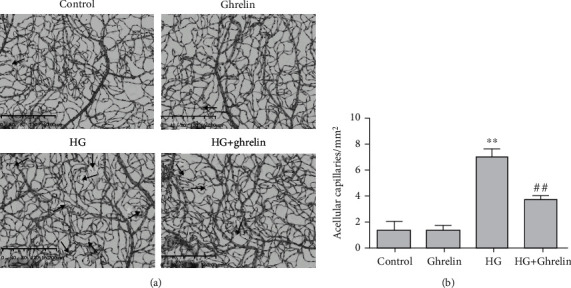
Retinal trypsin digestion assay: (a) black arrows indicate acellular vessels and (b) acellular vessels were quantified. ∗∗*P* < 0.05 vs. control group; ^##^*P* < 0.05 vs. HG group.

HG significantly increased the degenerate capillary numbers. Ghrelin had no effect on the mice without diabetes induction, and ghrelin treatment reduced the degenerate capillary numbers (Figure 7).

## 4. Discussion

The process of DR has been confirmed to be closely related to chronic oxidative stress and inflammation [12]. Oxidative stress is closely associated with diabetes and its complications, and it is the key factor in its occurrence and development [13]. Under the condition of diabetes, ROS can be generated through an enzymatic pathway and a non-enzymatic pathway. Excessive ROS can further cause retinal oxidative damage (e.g., aging and apoptosis), and the use of ROS inhibitors or modulators can improve the function of diabetic retina [14]. Oxidative stress is associated with inflammation, sufficient evidence has demonstrated that inflammation accompanies the progression of DR: the formation of inflammation and augmented concentration of ROS lead to retina degeneration [13, 15]. Therefore, protecting RPE cells and reducing the effect of pathogenic factors on the degeneration of retina play an important role in the prevention, treatment, and remission of DR.

Research has shown that the ROS signaling is required for NLRP3 inflammasome activation and the inhibition of mitochondrial ROS generation can inhibit the inflammatory activation of NLRP3 [16, 17]. The NLRP3 inflammasome is a member of the family of inflammatory corpuscles recently discovered and studied most widely. The activation of the NLRP3 inflammasome body triggers pro-caspase-1 activation to generate activated caspase-1, which promotes IL-1 *β* and IL-18 secretions [17]. The early response to DR is the activation of inflammatory signals in endothelial cells, the increase in ROS in mitochondria, and the subsequent activation of proinflammatory cytokines, such as TNF- *α*, IL-6, IL-18, MCP-1, and IL-1 *β* [18].

Excessive ROS production may induce oxidative stress, destroy mitochondrial function, and impair the antioxidant defense system. In this study, we measured SOD and MDA to assess cell redox status. HG suppressed SOD activity and increased MDA generation, treatment with ghrelin reversed this expression.

**Figure 8:**
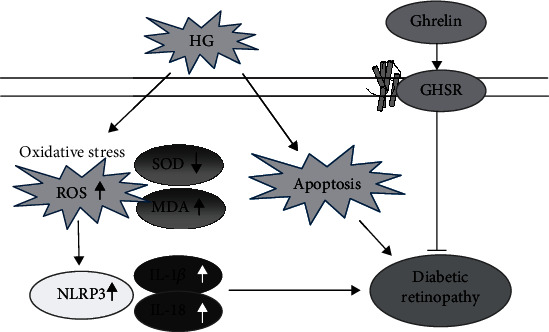
Summary of the effects of Ghrelin on HG­induced oxidative damage in RPE cells and the retina.

Scholars have tested the humoral components of DR patients in different stages, and the results show that the activation of NLRP3 inflammasome closely related to the pathogenesis of DR [19, 20]. IL-1*β* and IL-18 contribute to insulin resistance and islet *β*-cell damage in T2DM; they are classic pro-inflammatory cytokines of the IL-1 family. In this study, we observed that high glucose induced the production of oxygen radicals and apoptosis and upregulated the inflammatory cytokines IL-1*β* and IL-18. Ghrelin reversed the HG-induced increase in the NLRP3 inflammasome protein levels and decreased the IL-1*β* and IL-18 secretions (Figure 8).

Ghrelin is a 28-amino acid peptide and is the endogenous ligand of the growth hormone secretagogue receptor (GHSR). Its physiological functions include the inhibition of apoptosis and oxidative damage, regulation of metabolism, stimulation of growth hormone secretion, improvement of cardiovascular function, promotion of food intake and obesity, and immune anti-inflammatory [21, 22]. Ghrelin is secreted mainly by gastric fundus cells and acts on the hypothalamus and pituitary through peripheral circulation. In addition, ghrelin is expressed in the heart, pancreas, gastrointestinal tract, kidney, testis, ovary, placenta, and other tissues. It is also expressed in ocular tissues, such as the trabecular meshwork, ciliary body, choroid, iris, Müller cells, and retinal endothelial cells. Ghrelin in plasma can easily cross the blood–brain barrier; thus, it can also enter the eye tissue through the blood–eye barrier [23, 24]. The correlation between ghrelin and eye diseases has also been reported. Can found that exogenous ghrelin treatment could significantly reduce intraocular pressure in rats [25]. However, Eraslan et al. found that the expression level of ghrelin in aqueous humor of glaucoma patients was significantly lower than that of normal people [26]. Zhu et al. established the rat model of chronic glaucoma and found that ghrelin could activate the Akt mTOR signaling pathway by binding with GHSR, promote the survival of retinal ganglion cells, inhibit caspase-3 expression in retinal ganglion cells and Müller cells, and inhibit cell apoptosis, oxidative damage, inflammation, apoptosis, and other mechanisms [27].

In conclusion, using in vivo and in vitro studies, we demonstrated that ghrelin could protect ARPE-19 cells from HG-induced inflammation by decreasing the level of ROS production, inhibiting the NLRP3 inflammasome expression and IL-1*β* and IL-18 production, and reducing the apoptosis of retinal cells and retina histological damage in diabetic rats.

Currently, no report has been conducted yet on the toxicity of ghrelin, based on its safety and non-toxic characteristics, ghrelin may play a role in the treatment of ocular diseases involving DR.

## Data Availability

The data sets used and/or analyzed during the current study are available from the corresponding author on reasonable request.
